# Gallium-mediated siderophore quenching as an evolutionarily robust antibacterial treatment

**DOI:** 10.1093/emph/eou003

**Published:** 2014-01-30

**Authors:** Adin Ross-Gillespie, Michael Weigert, Sam P. Brown, Rolf Kümmerli

**Affiliations:** ^1^ Institute of Plant Biology, University of Zürich, Winterthurerstrasse 190, 8057 Zürich, Switzerland; ^2^Swiss Federal Institute of Aquatic Science and Technology (Eawag), Environmental Microbiology, Überlandstrasse 133, 8600 Dübendorf, Switzerland; ^3^Institute of Evolutionary Biology and Centre for Immunity, Infection and Evolution, University of Edinburgh, West Mains Road, Ashworth Laboratories, Edinburgh EH9 3JT, UK

**Keywords:** antivirulence therapy, public good quenching, resistance, experimental evolution, *Pseudomonas*

## Abstract

**Background and objectives:** Conventional antibiotics select strongly for resistance and are consequently losing efficacy worldwide. Extracellular quenching of shared virulence factors could represent a more promising strategy because (i) it reduces the available routes to resistance (as extracellular action precludes any mutations blocking a drug’s entry into cells or hastening its exit) and (ii) it weakens selection for resistance, as fitness benefits to emergent mutants are diluted across all cells in a cooperative collective. Here, we tested this hypothesis empirically.

**Methodology:** We used gallium to quench the iron-scavenging siderophores secreted and shared among pathogenic *Pseudomonas aeruginosa* bacteria, and quantitatively monitored its effects on growth *in vitro*. We assayed virulence in acute infections of caterpillar hosts (*Galleria mellonella*), and tracked resistance emergence over time using experimental evolution.

**Results:** Gallium strongly inhibited bacterial growth *in vitro*, primarily via its siderophore quenching activity. Moreover, bacterial siderophore production peaked at intermediate gallium concentrations, indicating additional metabolic costs in this range. *In vivo*, gallium attenuated virulence and growth—even more so than in infections with siderophore-deficient strains. Crucially, while resistance soon evolved against conventional antibiotic treatments, gallium treatments retained their efficacy over time.

**Conclusions:** Extracellular quenching of bacterial public goods could offer an effective and evolutionarily robust control strategy.

## Teaser

We tested the idea that antibacterial therapies targeting shared exoproducts would be more evolutionarily robust than regular antibiotics. Using gallium, we extracellularly quenched the iron-scavenging molecules shared among *Pseudomonas aeruginosa* bacteria, inhibiting their growth and virulence in caterpillars. During experimental evolution, gallium remained effective over time, while antibiotics did not.

## INTRODUCTION

Like all organisms, pathogens acquire genetic mutations, and, in time, even ‘pure’ cultures will inevitably come to harbor mutant lineages. Such genetic variability can make some pathogen variants less sensitive to therapeutic interventions than others, and under strong or sustained therapy, these resistant variants will have a selective advantage and will come to predominate over more susceptible variants. Consequently, the therapy will lose efficacy [1, 2]. To avoid this situation, we can try to prevent resistant variants from arising and/or from spreading [3]. To prevent resistance arising, we could attempt to reduce mutation supply, through limiting effective population size or by employing interventions with specialized modes of action where relatively few ‘routes to resistance’ are possible. To prevent spread, meanwhile, we must aim to minimize fitness differences across individual pathogens. Killing every individual, the conventional antibiotic strategy, could certainly quash fitness evenly, but this is difficult in practice and whenever incomplete gives resistant pathogens a strong relative fitness advantage. ‘Antivirulence’ treatments, meanwhile, ostensibly disarm but do not harm pathogens, such that resistant variants should benefit little relative to susceptibles [4]. However, traits that affect virulence but not fitness are rare, and the label ‘antivirulence’ is used liberally, even for interventions that yield substantial fitness differences among pathogens [4]. A final way to minimize fitness differences is to target pathogens’ collective traits, where costs and benefits are widely shared. For instance, many virulence-related bacterial exoproducts are also public goods (PGs) [5]. Under PG-quenching therapy, any mutations allowing PGs to build up again should benefit both resistant and susceptible individuals alike, which would hinder the spread of resistance [1, 6–8].

To illustrate why this matters, let’s consider a specific example. Quorum quenching (QQ), which disrupts the cell-to-cell communication [quorum sensing (QS)] [9] underlying a wide range of collectively expressed virulence traits, is a PG-targeting ‘antivirulence’ therapy regarded as a promising alternative to conventional bacteriocidal or bacteriostatic treatments [10, 11]. However, early enthusiasm for QQ has been tempered recently by reports that bacteria can quite readily evolve resistance to such treatments [12–14]. Set against our framework, this is unsurprising: first, QQ interventions frequently involve intracellular action, against which many potential resistance-conferring adaptations could arise (e.g. modified membrane properties to block a drug’s entry into a cell, or upregulated efflux pumps to hasten its exit [15]). Second, QS regulates not only PGs but also certain essential private goods [16], giving QQ resistants substantial personal benefits over susceptibles—and therefore a means to spread. For maximal evolutionary robustness, we need therapies where resistance mutations are unlikely to arise in the first place (e.g. extracellular action restricts potential routes to resistance) and are also unlikely to spread, because fitness differences between resistant and susceptible pathogens are minimized. The latter should be the case when collective traits are targeted, because fitness consequences are shared across many individuals. Of course, the extent and evenness of this sharing will depend on the relatedness and spatial structure of the pathogen population and the diffusive properties of the environment, and these factors would also need to be considered during therapy design [3].

In this study, we investigate—in a test case—the hypothesis that extracellular PG quenching is an effective and evolutionarily robust strategy for pathogen control. The PG trait we target is siderophores, important exoproducts whose regulation is not linked to any exclusively private goods. Siderophores are diffusible molecules with a high affinity for ferric iron (Fe^3+^) and are secreted by most bacteria to scavenge this important but generally bio-unavailable form of iron from their environment or, in the case of pathogens, from their host’s own iron-chelating compounds [17]. Once loaded with Fe^3+^, siderophores are taken up by producer cells—or other nearby individuals equipped with appropriate receptors—stripped of their iron, and secreted once again into the environment [18]. Although their primary function may be to scavenge iron, siderophores also bind, with varying success, several other metals [19, 20]. Among these, gallium is the closest mimic of iron. Ga^3+^ and Fe^3+^ ions have very similar ionic radii and binding propensities but, crucially, while Fe^3+^ reduces readily, Ga^3+^ does not [19]. Ga^3+^ therefore cannot replace iron as a co-factor in redox-dependent enzymes. We investigated the iron-mimicking effects of gallium on pyoverdine, the primary siderophore of *Pseudomonas aeruginosa* [21], a widespread opportunistic pathogen with a broad host range and, in humans, the cause of notoriously persistent infections in immune-compromised tissues, cystic fibrosis lungs and in association with implanted devices [22]. Pyoverdine, which plays an important role in such infections [23, 24], binds gallium at least as readily as iron, and gallium-bound pyoverdine is of no use to iron-starved cells [19, 20]. Thus, even without entering the cell, gallium can reduce *P. aeruginosa* growth and biofilm formation by quenching local stocks of secreted pyoverdine and choking off iron supply [19, 25].

Below, we report our investigations into (i) gallium’s *in vitro* interference with siderophore-mediated iron uptake and consequent effects on bacterial growth, (ii) gallium’s *in vivo* effects on virulence and in-host bacterial growth and (iii) the potential for bacteria to evolve resistance against gallium treatment.

## METHODOLOGY

### Strains and media

*Pseudomonas aeruginosa* strains featured in our experiments included the wild-type strain PAO1 (ATCC 15692), the siderophore knock-out mutants PAO1Δ*pvdD* and PAO1Δ*pvdDΔpchEF* [26], provided by P. Cornelis, Free University of Brussels, Belgium, as well as versions of the above strains constitutively expressing GFP (PAO1-*gfp*, PAO1ΔpvdD-*gfp*, chromosomal insertion: *att*Tn7::ptac-*gfp*), and a version of PAO1 with a *pvdA-gfp* reporter fusion (PAO1*pvdA-gfp*, chromosomal insertion: *att*B::*pvdA-gfp*) [27], provided by P. K. Singh, University of Washington, USA. We also used the Rhl-quorum-sensing deficient mutant PAO1Δ*rhlR*, provided by S. P. Diggle, University of Nottingham, UK. For overnight culturing, we used Luria Bertani (LB) medium, while for experimental assays we used CAA medium, supplemented with FeCl_3_ where indicated to manipulate iron availability. LB was obtained pre-mixed from Sigma-Aldrich, Switzerland. Our standard CAA medium contained 5 g l^−^^1^ casamino acids, 1.18 g l^−^^1^ K_2_HPO_4_*3H_2_O, 0.25 g l^−^^1^ MgSO_4_*7H_2_O, 100 μg ml^−^^1^ human-apo transferrin, 20 mM NaHCO_3_ and 25 mM HEPES buffer (all from Sigma-Aldrich).

### *In vitro *assays of growth and pyoverdine production

**Figure 1.**
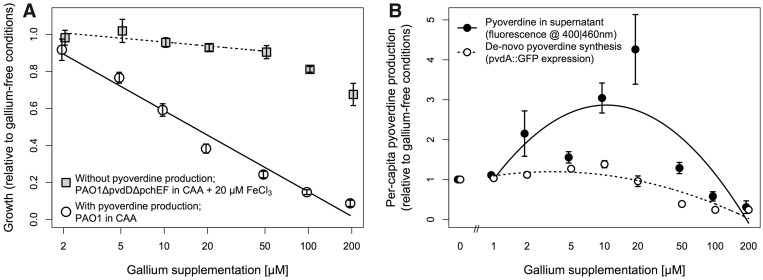
Gallium affects *P. aeruginosa*’s *in vitro* growth and siderophore production. (**A**) Gallium suppresses growth particularly when pyoverdine is present, as shown here by comparing conditions with and without its production. Symbols and bars indicate means and 95% CIs of integrals of spline curves fitted through 24 h growth trajectories (OD at 600 nm) of 12 replicate cultures. (**B**) Pyoverdine, assayed using complementary approaches, is in each case upregulated at intermediate gallium concentrations. Symbols and error bars represent means and SEs of five replicates. Measures of pyoverdine from supernatant (filled circles) or *pvdA* expression from cell fractions (open circles) are in each case scaled by cell density (OD at 600 nm).

Overnight LB cultures (37°C, 180 rpm), washed and standardized for cell density, were diluted to 10^−^^4^ then used to seed replicate cultures in CAA medium supplemented with Ga(NO_3_)_3_ (such that final Ga concentrations ranged from 0 to 200 μM), as well as complementary amounts of NaNO_3_ to balance nitrate levels across treatments, and 20 µM FeCl_3_ where iron-replete conditions were required (Fig. 1A). Growth assays were performed with 200 μl cultures in 96-well plates, for which optical density (OD) was tracked over 24 h at 37°C using a Tecan Infinite M-200 plate reader (Tecan Group Ltd., Switzerland), with 15 min read intervals preceded at each read by 10 s of agitation. To assay pyoverdine production, we first grew PAO1*pvdA-gfp* in 2 ml CAA static in 24-well plates in a 37°C incubator for 24 h, then centrifuged the cultures at 7000 rpm for 2 min to pellet the cells. From each culture, 200 µl of supernatant and, separately, the cell fraction resuspended in 200 µl 0.8% saline, were transferred to a new 96-well plate and assayed for OD at 600 nm and fluorescence (GFP in cell fraction: ex|em = 488|520 nm; pyoverdine in supernatant: 400|460 nm) [28]. Both fluorescence measures were standardized by OD at 600 nm. In a series of side experiments, we investigated potential biases associated with the use of optical measures as proxies for pyoverdine production (Supplementary Fig. S1). Data presented in Fig. 1B are corrected for these biases. 

### Experimental infections

Infection assays were performed with final instar *Galleria mellonella* larvae, purchased from a local supplier, standardized for mass and general condition and stored at 4°C until use (within 3 days). A Hamilton precision syringe was used to deliver 10 µl inocula via a sterile 26s gauge needle introduced sub-dermally to a surface-sterilized area between the last pair of prolegs. Inoculations contained Ga(NO_3_)_3_ diluted to different concentrations in 0.8% saline, with complementary concentrations of NaNO_3_, and, where specified, bacteria from overnight LB cultures (37°C, 180 rpm), standardized for cell density and diluted such that each 10 µl inocula contained ∼25 CFU (*post hoc* counts of 12 inocula plated out to LB agar gave 95% CI of 19.41–31.76). Specifically, we tested the following Ga(NO_3_)_3_ concentrations: 2.5, 10 and 50 μM (‘LOW’; pooled together since their resulting virulence curves were not significantly different from one another), 500 μM (‘MED’) and 2500 μM (‘HIGH’). Our ‘Gallium only’ treatment comprised various concentrations between 2.5 and 2500 μM, which again we pooled for statistical analyses because of similar effects on survival. Post-injection, larvae were placed individually in randomly allocated wells of 24-well plates and incubated at 37°C. Survival was monitored hourly between 10 and 24 h, and larvae were considered dead once they no longer responded to tactile stimulation. Any larvae that began to pupate while under observation or died within the first 10 h post-injection (i.e. as a result of handling) were excluded from analyses (*n* = 23, 3.6%). To assay *in vivo* bacterial growth, we prepared our inocula with strains engineered to constitutively express GFP (see above), having previously established that constitutively expressed GFP signal could provide a reliable correlate of bacterial density under the conditions of this infection model (Supplementary Fig. S2). In each of six separate experimental blocks, and at each of four discrete timepoints, 3–4 randomly selected larvae per treatment were flash-frozen in liquid N_2_ and manually powderized. Powdered larval homogenates were resuspended in 1 ml sterile H_2_O, vigorously shaken and then centrifuged at 7000 rpm for 2 min, whereafter the sample segregated into discrete phases. About 200 µl of the water-soluble liquid phase was extracted and assayed for GFP-fluorescent signal relative to control replicates (saline-injected larvae), using a Tecan Infinite M-200 plate reader. Given total larval volumes of ∼1 ml, and assuming that ∼20% of this volume might be hemolymph accessible to particles diffusing from a single injection site during the course of an acute infection, we estimate that inocula gallium concentrations of 2.5–2500 μM would translate to in-host gallium concentrations of roughly ∼0.05 to ∼50 μM.

### Experimental evolution

We compared the growth inhibitory effects of gallium versus the aminoglycoside, gentamicin (Gm), and the fluoroquinolone, ciprofloxacin (Cp)—two of several antibiotics recommended for clinical use against *P. aeruginosa* [29]. Concentrations were calibrated such that they reduced growth integrals over the initial 24 h to ≤1/3 that of untreated PAO1 WT cultures under the same growth conditions. For each of 12 days, a 96-well plate was prepared, comprising replicate 198 μl volumes of iron-limited CAA medium supplemented, according to a randomized layout scheme, with gallium, antibiotics or an equivalent volume of saline (see key in Fig. 3 for details of treatments used and their respective sample sizes). Day 1 cultures were initiated with 2 μl aliquots of a 10^−^^3^ diluted overnight LB culture of PAO1 WT (37°C, 180 rpm), while for subsequent days, fresh plates were inoculated with 10 μl of undiluted culture from the corresponding wells of the previous day’s plate, directly after it completed its growth cycle. Plates were incubated at 37°C, and cell density and pyoverdine fluorescence measures were recorded at 15 min intervals (with 10 s initial shaking) using a Tecan Infinite M-200 plate reader.

### Endpoint phenotypic assays

Prior observations [30] and our own reasoning (see Table 1) suggested that pyoverdine and pyocyanin could both affect the costs and benefits of iron uptake under gallium treatment. Anticipating that the experimental evolution described above might have induced changes in these traits, we performed phenotypic assays to compare cultures of our ancestral PAO1 WT, its descendent lines experimentally evolved in CAA with or without supplementation with 20 μM Ga, and also two knock-out mutant strains which served as negative controls: PAO1Δ*pvdD* (deficient for pyoverdine production) and PAO1Δ*rhlR* (deficient for the Rhl-quorum-sensing system which regulates pyocyanin production [31]). Specifically, we inoculated 2 ml volumes of growth medium (either LB or CAA) with 20 μl of 10^−^^3^ diluted overnight LB culture and incubated at 37°C in static conditions. After 24 h, we measured OD 600, centrifuged at 7000 rpm for 2 min, then extracted 200 μl aliquots of supernatant and assayed these for growth (OD at 600 nm) and levels of pyocyanin (using OD at 691 nm) [32] and pyoverdine (fluorescence at 400|460 nm), using a Tecan Infinite M-200 plate reader.

### Statistical analyses

All analyses were performed using R 3.0.0 [33]. Spline curves were fitted to time course growth data using the ‘grofit’ package [34]. Survival analyses were performed using the Surv package [35]. Although in the main text we compared survival curves using parametric Weibull models, we also repeated all analyses using Cox proportional hazards regressions, and obtained qualitatively comparable results in all cases.

## RESULTS

In *in vitro* assays, we found that gallium strongly inhibited bacterial growth, and that the inhibitory effects were mediated primarily via gallium’s extracellular quenching activity and not because gallium is toxic *per se* (Fig. 1A). When siderophores were required and could be produced, increasing gallium concentration was associated with a steep decline in growth (slope ± SE of regression with log_10_[Ga]: −0.435 ± 0.011, *t* = −38.04, *P* < 0.001). In contrast, when siderophores were not required and not produced, gallium only weakly affected growth (slope ± SE: −0.067 ± 0.019, 95% CI for drop = [2.91–15.86%]; difference in slopes 0.368 ± 0.022, *F*_1,__140_ = 276.41, *P* < 0.001)—particularly over the range of concentrations up to and including 50 μM, which correspond to the concentrations likely experienced in our *in vivo* experiments (see below).

It has been suggested that as the benefit of pyoverdine production drops, bacteria should gradually scale back their investment in this trait [19]. On the other hand, it has also been shown that pyoverdine production is upregulated in response to more stringent iron limitation [28], as presumably induced by gallium. Here, we saw a combination of these two regulatory effects, with investment to replace quenched pyoverdine actually increasing from low to intermediate gallium supplementation levels and cessation becoming evident only at higher concentrations (Fig. 1B; ANOVA comparison of quadratic versus linear fits: *F*_1,__36_ > 15, *P* < 0.001 in each case).

**Figure 2.**
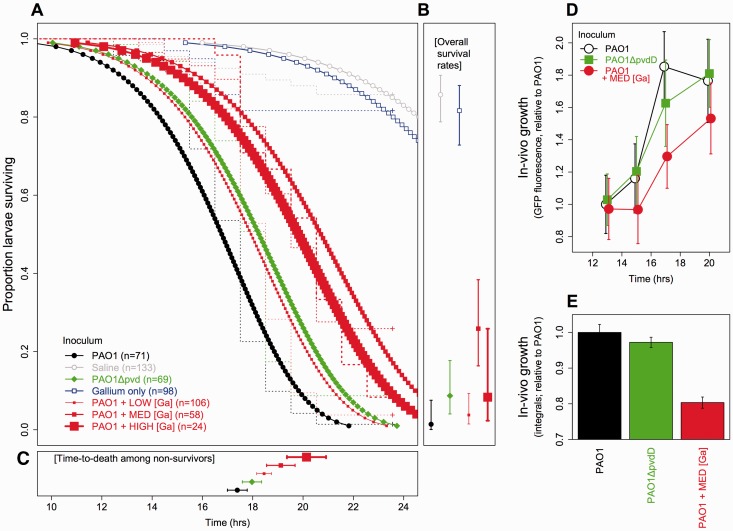
Gallium attenuates *P. aeruginosa* virulence and growth in *G. mellonella* larvae. (**A–C**) Virulence across treatments, as Kaplan–Meier (stepped lines) and Weibull (smoothed lines) survival curves; proportion surviving (with 95% binomial CIs); and time-to-death (means and 95% CIs). We estimate that inocula with ‘LOW’ (2.5–50 μM), ‘MED’ (500 μM) or ‘HIGH’ (2500 μM) concentrations of Ga(NO_3_)_3_ gave in-host concentrations of ∼0.05 to ∼50 μM (see ‘Methodology’ section). (**D**) Bacterial density *in vivo* (GFP signal in host homogenate; means and 95% CIs from ∼24 larvae) corrected against saline-injected controls and scaled relative to PAO1 at 13 h. (**E**) Mean and 95% CIs of bacterial growth integrals derived from bootstrap replicate time series (24 replicate splines) from (D)

Given our *in vitro* observations of gallium’s effects on growth and pyoverdine production, we expected it to affect virulence and bacterial fitness *in vivo* too. We tested this in experimental infections of greater waxmoth larvae (*G. mellonella*). Gallium-supplemented *P. aeruginosa* infections indeed showed significantly attenuated virulence compared with non-supplemented infections (Fig. 2A–C; Weibull curve comparison: *z* = 3.10–7.82, *P* < 0.001 in all cases). Notably, infections supplemented with medium and high concentrations of gallium (corresponding to the intermediate gallium concentration used in the *in vitro* assays, see ‘Methodology’ section) were significantly less virulent (*z* = 4.96 and 2.39, *P* < 0.05 in both cases) than infections with PAO1Δ*pvdD*, a mutant defective for pyoverdine production that itself showed attenuated virulence versus PAO1 (*z* = 3.49, *P* < 0.001). Gallium alone appeared to have little effect on hosts, with levels of virulence not significantly different from those seen in saline-injected controls (Fig. 2A: survival curve comparison: *z* = −0.93, *P* = 0.35; Fig. 2B: pairwise proportion tests for survival rates: ${\text{X}}_{1}^{2}$ = 0.43, *P* = 0.51). Bacterial growth *in vivo* was also significantly reduced by gallium (Fig. 2D and E). Growth integrals were lower in gallium-supplemented larvae than in WT-injected larvae (Fig. 2E; Tukey’s 95% CIs for the difference: 16.21–21.44%, *t* = 17.24, *P* < 0.001) and, moreover, lower than in larvae injected with the siderophore-defective mutant, PA01Δ*pvdD* (Fig. 2E; Tukey’s 95% CI = 13.58–18.97%, *t* = 14.45, *P* < 0.001). 

**Figure 3.**
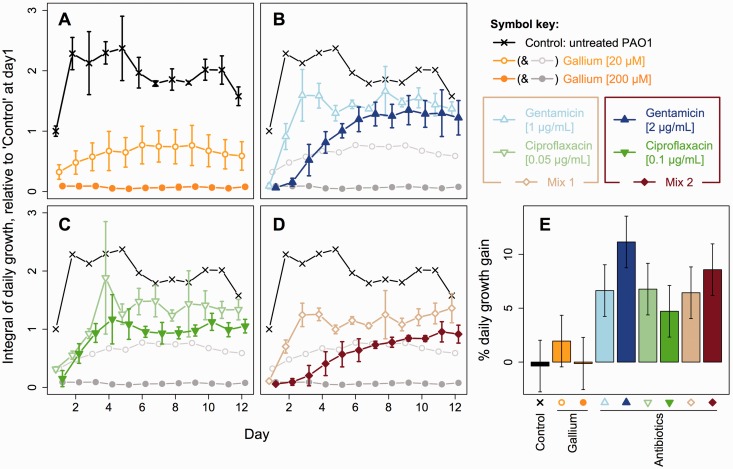
Evolutionary potential for resistance against gallium treatment. (**A–D**) Over the course of experimental evolution, daily growth integrals for cultures treated with various antibiotics rose significantly, while the growth of gallium treated cultures did not. (**E**) Slope coefficients for linear fits through data in (A–D), expressed as % of growth of control at Day 1. In all cases, symbols and error bars show means and 95% CIs of six replicate cultures

To investigate empirically the general potential for resistance against gallium, we performed experimental evolution with serial batch cultures, comparing *P. aeruginosa* exposed to gallium versus several single- and mixed-antibiotic regimes (Fig. 3A–E). At first, all treatments were strongly refractory to growth, showing 24 h growth integrals no more than a third those of untreated controls (range: 5.8–32.3%). Over the course of a 12-day experiment (a therapy duration that matches clinical standards), however, the growth in all antibiotic treatments increased significantly (Fig. 3E; *H*_0_ slopes = 0: Cp1: *t* = 5.54, Cp2: *t* = 3.86, Gm1: *t* = 5.43, Gm2: *t* = 9.12, Mix1: *t* = 5.26, Mix2: *t* = 7.02; *P* < 0.001 in each case), and by the final timepoint their growth integrals were comparable to those of the untreated controls at the start of the experiment (Fig. 3A–D). Gallium-treated cultures, meanwhile, like the untreated control, did not show a significant trend toward higher growth (Fig. 3E; *H*_0_ slopes = 0; *t* = −0.30, *P* = 0.76; *t* = 1.60, *P* = 0.11 and *t* = −0.11, *P* = 0.91 for control, Ga1 and Ga2, respectively). 

Per-capita pyoverdine output was generally steady over the course of experimental evolution [Supplementary Fig. S3: *H*_0_ slopes = 0: control: *z* = 0.56, *P* = 0.58; Ga1: *z* = 0.45, *P* = 0.65; all antibiotic treatments pooled (Day 1 excluded): *z* = 0.83, *P* = 0.41], with that of the 20 μM gallium treatment consistently around 2-fold higher than either control or antibiotic-treated cultures (95% CIs for fold-difference were 1.86–2.13 versus control, and 1.96–2.24 versus pooled antibiotic treatments).

**Figure 4.**
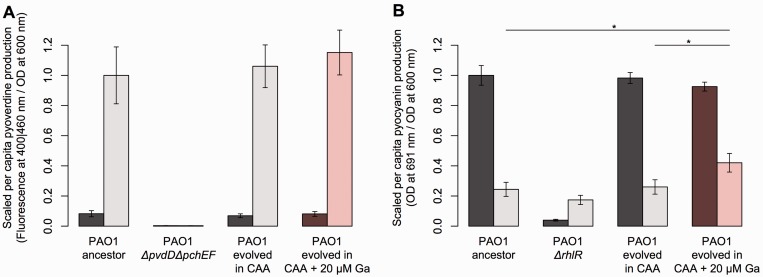
Resistance-related phenotypic changes following experimental evolution under gallium treatment. Pyoverdine (**A**) and pyocyanin (**B**) production under standardized test conditions (dark bars = LB medium, light bars = CAA medium) of ancestral PAO1, knock-out strains (i.e. negative controls), control lines (evolved without gallium) and gallium-selected lines. Pyoverdine measures are scaled to that of PAO1 in CAA, whereas pyocyanin is scaled to that of PAO1 in LB. Asterisks indicate cases where Ga-selected lines were significantly different from their ancestor and unexposed control lines. Error bars give 95% CIs of 3–6 replicates

In the endpoint phenotypic assays performed under standardized test conditions (CAA and LB media), lines evolved in the Ga1 treatment showed no significant change in pyoverdine production (Fig. 4A) relative to their ancestor (CAA: *t* = 0.81, *P* = 0.43; LB: *t* = 0.08, *P* = 0.94) or to lines evolved under control conditions (CAA: *t* = −0.49, *P* = 0.63; LB: *t* = 0.95, *P* = 0.36), suggesting that the high pyoverdine output seen during experimental evolution was predominantly a plastic response to gallium (see Fig. 1B). In contrast, the production of pyocyanin did appear to be elevated in the Ga1 endpoint isolates (Fig. 4B) in CAA medium (versus ancestor: *t* = 3.40, *P* = 0.004; versus control: *t* = 3.09, *P* = 0.008) but not in LB medium (versus ancestor: *t* = 1.69, *P* = 0.12; versus control: *t* = 1.56, *P* = 0.15). 

## DISCUSSION

The results reported above indicate that gallium inhibits *P. aeruginosa* growth primarily through extracellular interference with its primary siderophore, pyoverdine (Fig. 1A); that this growth inhibition occurs in an infection context too (Fig. 2D and E), along with a significant reduction in virulence (Fig. 2A–C); and that resistance to gallium treatments does not evolve easily—at least not in comparison to two conventional antibiotics we tested (Fig. 3).

For gallium to be both optimally effective and evolutionarily robust as an antibacterial agent, an appropriately calibrated dose will be key. At lower concentrations, efficacy should initially increase with dose, but at too high concentrations, gallium may increasingly transit across the cell membrane and begin to interfere directly with iron metabolism, causing general toxicity to bacteria and host cells alike (Fig. 1A; [36]). Here, fitness costs are imposed intracellularly at the individual cell level, and not extracellularly at the level of the collective, which would take us back to a classic antibiotic scenario, with more potential ‘routes to resistance’ and greater potential for steep fitness gradients among individual cells. At sub-toxic levels, meanwhile, where gallium acts primarily through siderophore-quenching, resistance should evolve less readily. Furthermore, we saw that the costs and benefits of siderophore investment itself are also non-linear functions of gallium concentration, owing to the existence of a regulatory ‘trap’. Specifically, intermediate concentrations of gallium induced the highest levels of replacement pyoverdine production in bacteria (Fig. 1B), adding further metabolic stress to increasingly iron-limited cells. Our *in vivo* results, which showed that gallium can suppress virulence to levels beyond those seen in pyoverdine-deficient strains (Fig. 2A–C), are consistent with the interpretation that an appropriate dose of gallium not only restricts bacterial iron uptake but can also impose a costly metabolic burden. Given our understanding of the regulation of pyoverdine production, this hump-shaped association between pyoverdine investment and gallium is to be expected. Positive feedback occurs when incoming Fe^3+^-bound siderophores act via the receptor FpvA and the anti-sigma factor FpvR to activate membrane-bound iron-starvation sigma factor PvdS [37]. High cytoplasmic Fe^2+^ levels, meanwhile, can generate negative feedback. In this case, the Fe^2+^ induces Fur (ferric uptake regulator)-mediated repression of *pvdS* [38]. At low gallium concentrations, iron uptake into the cell is steady, so negative feedback keeps pyoverdine production at some intermediate level, while at mid-range gallium concentrations, iron uptake becomes increasingly restricted, leading to steady positive feedback, but weaker negative feedback, and consequently, pyoverdine production increases. Finally, at high concentrations, iron uptake may be so severely restricted that the positive feedback loop fails, and pyoverdine production stalls completely. Exploiting metabolic ‘traps’ such as this could significantly increase the effectiveness of treatments, but requires that the associated regulatory networks should be left intact and functional. This raises another point in favor of extracellular quenching strategies, as opposed to, say, intracellular-mediated deactivation of entire molecular pathways.

To what extent should gallium’s antibacterial activity be evolutionarily robust? In our selection experiment (Figs 3 and 4A), we saw little evidence of adaptation to gallium, although perhaps we can still predict what sort of phenotypic changes could conceivably confer resistance against gallium-mediated siderophore quenching, and under which conditions such adaptions could spread. Below, we consider several potential evolutionary responses, which are discussed further in Table 1.

First, let’s consider pyoverdine loss-of-function mutants, which are known to arise readily under iron limited conditions [39–41]. In co-infection with siderophore producers, non-producing mutants could act as cheats—no longer investing in the PG yet still benefiting from the investment of nearby ancestors [5]. Even as opportunities to cheat dwindled, such mutants could continue to spread, since, disadvantaged as they would be with respect to autonomous iron acquisition, they would at the same time be freed of the substantial extra metabolic burden of pyoverdine production under gallium regimes (see Fig. 1B). Depending on specific conditions within host tissues, the net fitness of non-producers could be not far off that of pyoverdine producers (Fig. 2E), so the mutants could potentially come to occupy a substantial share of the population. We saw no significant change in mean pyoverdine production in strains evolved under gallium (Fig. 4A), suggesting that cheats did not gain prominence in these cultures. However, certain individual lines (three antibiotic lines and one Ga1 line) went extinct during the course of the experimental evolution, and this extinction was in each case accompanied by a crash in per capita pyoverdine production levels (Supplementary Fig. S3), which would be consistent with a scenario of siderophore-non-producing cheats spreading in these cultures. In any event, the rise of such mutants should still lead to less virulent infections (Fig. 2A–C; [42–44]).

Alternative scenarios for evolutionary responses to gallium treatment could involve modifying pyoverdine to have substantially greater affinity for Fe^3+^ than for Ga^3+^, or switching to ‘backup’ siderophores relatively less susceptible to gallium (Table 1). Such mutations could conceivably arise but in each scenario we would expect attendant selection for the mutation to be relatively weak because, as PGs, these alternative or modified siderophores’ benefits would still be accessible to all cells within diffusion range, including those lacking the novel mutation. In addition, gallium and iron remain fundamentally similar in their physical properties, such that gallium will still bind—to some extent at least—any modified siderophore. 

**Table 1.** How likely is resistance against gallium-mediated pyoverdine quenching?

Mutant phenotype	Why resistant?	Likelihood for mutant to arise	Likelihood for mutant to spread
Pyoverdine production reduced or shut down.	No true resistance, as virulence is only partly restored. However, mutants could avoid being ‘trapped’ into high pyoverdine production (Fig. 1B), which can be a substantial fitness drain (Fig. 2E).	High	Low
		Pyoverdine-negative mutants arise readily [39, 41].	In mixed cultures, gallium reduces total population density and the effective group size at which pyoverdine can be shared, and these effects both disfavor the mutant [45, 46].
Pyoverdine modified to bind iron with greater specificity.	Iron uptake efficiency, and hence growth, should improve.	Low Pyoverdine has already evolved high iron specificity [20]. Further improvements are unlikely. Ga^3+^ and Fe^3+^ remain fundamentally very similar in binding behavior.	Low Pyoverdine molecules are shared across the local community [47], so producers of the novel and the ancestral pyoverdine types would benefit similarly.
Regulatory shift from producing pyoverdine to producing pyochelin, a secondary siderophore normally deployed in less iron-limited conditions.	Although pyochelin is generally a less effective siderophore than pyoverdine, this strategy could be advantageous under extreme conditions (e.g. in the presence of gallium).	High	Low Like pyoverdine, pyochelin is also a shared trait, so benefits would go to non-mutants too. Gallium can quench pyochelin too, and so it still inhibits iron uptake [49].
		Regulatory mechanisms already exist to facilitate facultative switching between siderophore types in response to changing iron stress [48]. Mutations that alter this switch could probably arise easily.	
Own pyoverdine production reduced + specialization to use heterologous siderophores from other co-infecting species.	Ceasing pyoverdine production would reduce personal costs, and heterologous siderophores could offer compensatory benefits.	Low	Low Most siderophores (e.g. desferrioxamine) are still prone to bind gallium [51]. Wild-type *P. aeruginosa* can also facultatively switch to heterologous siderophore use whenever such siderophores become available [50].
		Although *P. aeruginosa* can already take up heterologous siderophores (e.g. enterobactin, desferrioxamine) [50], this route would require co-infection with a bacterium that produces an accessible siderophore.	
Own pyoverdine production reduced + specialization to take up iron directly from the host.	Ceasing pyoverdine production would reduce personal costs, while iron from other sources could offer compensatory benefits.	High	Low Some host iron chelators might also bind gallium (e.g. citrate). Wild-type *P. aeruginosa* can also facultatively switch to alternative uptake mechanisms when such sources become available [50].
		*P. aeruginosa* already possesses the means to take up iron in various forms [50], including when it is in complex with hosts’ iron chelators. A simple switch in a regulatory pathway might be all that is required.	
Upregulated production of reducing agents (e.g. pyocyanin), which extracellularly reduce ferric to ferrous iron.	Reducing agents increase availability of the more soluble ferrous form of iron (Fe^2+^), which can be taken up without the need for siderophores.	High	Low Increased production of a metabolite would induce extra costs. Like pyoverdine, pyocyanin is also a shared trait, so benefits (in the form of ferrous iron) would go to non-mutants too.
		Upregulation of an already existing trait could be achieved easily [30].	

Here, we consider various mutant phenotypes that could putatively confer resistance, and propose hypotheses regarding the likelihood of emergence and spread in each case.

Further possible evolutionary responses could involve mutants that specialize in the direct uptake of Fe^3+^-containing compounds produced by other competing microbes (i.e. inter-specific cheats), or present as chelators in the host tissues. Such mutations are also conceivable, given that bacteria already possess a diversity of iron-uptake machineries [50]. However, considering that gallium can displace Fe^3+^ from other compounds too, it is not clear that such strategies would offer any clear advantages over siderophore-mediated uptake.

Finally, bacteria could potentially sidestep their dependence on the Fe^3+^ form of iron (prevalent under oxygen replete and neutral pH conditions) by altering their environment to increase the extracellular availability of the more bio-available Fe^2+^ ions. Indeed, overproducers of pyocyanin, a redox-active metabolite, have recently been reported to be refractory to gallium [30], and in our own experiments, we did see a weak but significant mean increase in pyocyanin production under certain conditions among cultures evolved under gallium treatment (Fig. 4B). However, such metabolites are themselves PGs, so the spread of over-producers could be constrained in due course by the free-loading behavior of variants that produce less, yet still benefit by the increased availability of Fe^2+^ ions.

In our experimental infections, we observed that gallium supplementation reduced both the virulence and the in-host fitness of *P. aeruginosa* (Fig. 2). However, pathogen fitness and virulence will not always be strongly positively correlated [43, 52]. For example, we showed that intermediate gallium induced overexpression of pyoverdine (Fig. 1B), and in some contexts, this could potentially lead to higher virulence, given that pyoverdine production is linked to certain other virulence factors [53, 54]. Indeed, while gallium is generally known to reduce virulence [19], one recent study [55] showed that in very dense cultures, gallium supplementation actually upregulated production of certain virulence factors. Thus, while gallium represents a promising way to reduce bacterial load, its overall effectiveness in reducing damage to a host will, as always, depend also on the particular characteristics of the host and its interaction with the pathogen.

## CONCLUSIONS AND IMPLICATIONS

Gallium has seen application in medical contexts for years (e.g. as an anti-cancer drug [56]) and has previously been proposed, and tested, as a treatment against bacterial infections [19, 25, 57, 58]. Gallium can be directly toxic at high concentrations, but here, working with concentrations below this toxic range, we have focused on its capacity to indirectly affect bacteria through disruption of siderophore-mediated iron uptake. Specifically, gallium quenches siderophores extracellularly, starving cells of iron and pushing them into a metabolically costly regulatory trap from which there seems to be little scope for evolutionary escape. In light of our results, we contend that this approach—and more generally the extracellular targeting of PGs—could curb microbial virulence in an evolutionarily robust manner, and therefore represents a promising alternative to our dwindling succession of traditional antibiotics [59–61].

## SUPPLEMENTARY DATA

Supplementary data are available at *EMPH* online and at the Dryad depository: doi:10.5061/dryad.8kk36.

## Supplementary Material

Supplementary Data
